# Case report: First case of pemetrexed plus cisplatin-induced immune hemolytic anemia in a patient with lung adenocarcinoma

**DOI:** 10.3389/fmed.2022.917485

**Published:** 2022-08-25

**Authors:** Hongkai Lu, Na Wang, Peng Wang, Haolin Zhang, Ru Zhao, Hongju Liu, Xirong He, Zeya Liu, Yue Chang, Yongtong Cao, Shiyao Wang

**Affiliations:** ^1^Department of Blood Transfusion, China-Japan Friendship Hospital, Beijing, China; ^2^Department of Blood Transfusion, Peking University First Hospital, Beijing, China; ^3^Faculty of Environment and Life, Beijing University of Technology, Beijing, China; ^4^Clinical Laboratory, United Family Women's and Children's Hospital, Beijing, China; ^5^Clinical Laboratory, China-Japan Friendship Hospital, Beijing, China; ^6^Department of Pulmonary and Critical Care Medicine, National Center for Respiratory Medicine, National Clinical Research Center for Respiratory Diseases, China-Japan Friendship Hospital, Beijing, China; ^7^Institute of Respiratory Medicine, Chinese Academy of Medical Sciences, Beijing, China

**Keywords:** pemetrexed, cisplatin, complement-dependent antibody, non-immunologic protein adsorption, drug-induced immune hemolytic anemia

## Abstract

**Background:**

Drug-induced immune hemolytic anemia (DIIHA) is a rare but potentially life-threatening drug-related complication. There are no previous reports of pemetrexed plus cisplatin as first-line chemotherapy for non-small cell lung cancer, resulting in DIIHA.

**Case presentation:**

In this report, a patient with advanced-stage lung adenocarcinoma developed severe immune hemolytic anemia 21 days after pemetrexed plus cisplatin chemotherapy. Laboratory findings showed severe hemolysis, including a rapid decrease in hemoglobin (HGB) and an elevated level of reticulocytes (Rets), indirect bilirubin (IBIL), and lactate dehydrogenase (LDH). A workup for the possibility of DIIHA was performed, including a direct antiglobulin test (DAT), a test in the presence of the soluble drug, and a drug-treated red blood cell (RBC) test. It showed a strongly positive (3+) result for anti-C3d but not for anti-immunoglobin G (IgG) in DAT. Enzyme-treated RBCs reacted weakly with the patient's serum and pemetrexed when complement was added. In addition, the patient's serum and normal sera were reactive with cisplatin-treated RBCs. However, eluates from the patient's RBCs and diluted normal sera were non-reactive with cisplatin-coated RBCs. Untreated and enzyme-treated RBCs reacted with the patient's serum in the presence of soluble cisplatin. *In vitro* serological tests suggested that complement-dependent pemetrexed antibodies and cisplatin-associated non-immunologic protein adsorption (NIPA) might combine to cause immune hemolytic anemia. The patient's anemia gradually recovered when pemetrexed and cisplatin were discontinued.

**Conclusion:**

This rare case demonstrated that complement-dependent pemetrexed antibodies and cisplatin-associated NIPA might occur simultaneously in a patient with DIIHA.

## Introduction

Drug-induced immune hemolytic anemia (DIIHA) is a rare but severe complication related to drug application, leading to serious adverse outcomes. The incidence of DIIHA is ~1–4/ million/year (1–3). However, the incidence is likely to be underestimated because many cases are often overlooked, often misdiagnosed, and often diagnosed as warm autoimmune hemolytic anemia (4–6). In cases of suspected DIIHA, several aspects should be considered: medical history, signs, types of drug, timing of drug initiation, the measure of administration, hemolytic features (extravascular or intravascular hemolysis), and comorbidities (6).

Serologic testing for associated drug-induced antibodies is very useful for verifying and differentiating the diagnosis of DIIHA. Drugs that interact with the immune system can elicit the formation of red blood cell (RBC) antibodies, including drug-dependent and drug-independent. Drug-dependent antibodies reacting *in vitro* only in the presence of the drug either bind to the membrane of RBCs or dissolve in the patient's serum. These antibodies are directed against drugs alone or a combination of drugs and RBC membrane antigen. In contrast, drug-independent antibodies against RBCs can be detected *in vitro* by the direct antiglobulin test (DAT) and the indirect antiglobulin test (IAT) in the absence of drugs (2, 7). In addition to drug-induced antibodies and autoantibodies, non-immunological adsorption of proteins (NIPA) of some drugs is also responsible for DIIHA (8, 9). These drugs may modify the RBC membrane, and then, the proteins are non-specifically adsorbed on the modified membrane (10). NIPA can cause positive DATs and IATs, which may confound the interpretation of tests that measure drug-induced antibodies.

Approximately 140 drugs were reported to induce DIIHA (11), of which chemotherapeutic drugs (mainly platinum-based agents) account for a significant proportion (5). In this study, we report a patient who developed DIIHA after receiving intravenous infusion of pemetrexed plus cisplatin chemotherapy for lung adenocarcinoma.

## Case presentation

**Figure 1:**
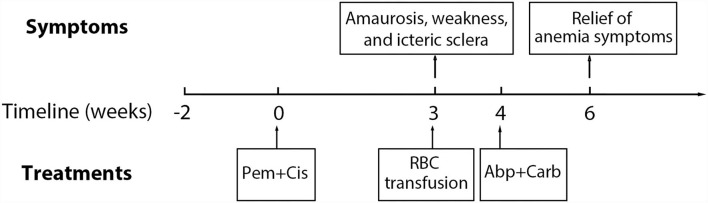
Timeline of patient's clinical course. Pem, pemetrexed; Cis, cisplatin; Abp, albumin-bound paclitaxel; Carb, carboplatin; RBC, red blood cells.

A 65-year-old male patient was admitted to the hospital with a chief complaint of cough and hemoptysis for 2 months. Cheat computed tomography (CT) suggested a mass in the right lung with mediastinal adenopathy. The diagnosis of lung adenocarcinoma (cT_4_N_2_M_0_, Stage IIIB) was established based on histopathology obtained from a transbronchial biopsy and endobronchial ultrasound guided transbronchial needle aspiration. The patient then underwent the first cycle of pemetrexed (500 mg/m^2^, Day 1) combined with cisplatin (75 mg/m^2^, Day 1) chemotherapy on 7 November 2021, after preparation with folic acid, vitamin B12, and glucocorticoids. On 26 November 2021, the patient developed amaurosis, weakness, and icteric sclera and was admitted to the hospital again. Laboratory tests showed a rapid drop in hemoglobin (HGB) and elevated levels of reticulocytes (Rets), indirect bilirubin (IBIL), and lactate dehydrogenase (LDH). There were no schistocytes in the peripheral blood smear. White blood cell count and platelet count were normal. A possible diagnosis of bone marrow suppression or thrombotic thrombocytopenic purpura has been ruled out. Chest CT showed no signs of lung parenchymal hemorrhage. Repeated occult blood tests of stool were negative. There was no evidence of bleeding from the lung or gastrointestinal tract. Additionally, antinuclear antibodies were negative. Peripheral CD55^−^CD99^−^ monoclonal RBCs were not detected by flow cytometry assay, which ruled out the diagnosis of paroxysmal nocturnal hemoglobinuria. DAT showed a strong positivity with anti-C3d (3+) but not with anti-immunoglobin G (IgG). The patient's medication history and main symptoms are shown in Figure 1 (Timeline of patient's clinical course). The detailed results of related laboratory tests of blood samples collected at 2 weeks (25 October 2021) pre-chemotherapy and 3 (27 November 2021), 4 (4 December 2021), 6 (18 December 2021), 8 (31 December 2021), 11 (21 January 2022), and 14 weeks (11 February 2022) post-chemotherapy are listed in Table 1.

**Table 1:** Laboratory test results of the patient before and after pemetrexed plus cisplatin chemotherapy.

**Detection items**	**2 weeks pre- chemotherapy**	**3 weeks post- chemotherapy**	**4 weeks post- chemotherapy**	**6 weeks post- chemotherapy**	**8 weeks post- chemotherapy**	**11 weeks post- chemotherapy**	**14 weeks post- chemotherapy**	**Reference range**
Hemoglobin (g/L)	127.00	68.00	69.00	86.00	104.00	111.00	91.00	130.00–175.00
RBC count (×10^12^)	4.54	2.30	1.79	1.58	3.53	3.65	3.11	4.30–5.80
MCV (fL)	85.10	82.20	97.20	94.30	92.10	89.30	87.50	82.00–100.00
MCH (pg)	28.00	29.60	38.50	54.40	29.50	30.40	29.30	27.00–34.00
MCHC (g/L)	328.00	360.00	397.00	577.00	320.00	340.00	335.00	316.00–354.00
Reticulocyte proportion (%)	NT	2.93	9.58	1.25	NT	NT	NT	0.50–1.50
Platelet count (×10^9^)	274.00	781.00	445.00	310.00	185.00	192.00	139.00	100.00–350.00
WBC count (×10^9^)	7.24	21.77	13.14	3.83	2.75	3.23	4.27	3.50–9.50
Total bilirubin (μmol/L)	6.84	36.21	9.31	8.97	NT	10.05	8.39	5.00–21.00
Indirect bilirubin (μmol/L)	4.56	27.96	5.86	6.90	NT	9.12	6.50	3.40–17.00
LDH (IU/L)	185.00	593.00	318.00	245.00	NT	NT	210.00	100.00–250.00
Folic acid (nmol/L)	NT	19.42	NT	NT	NT	NT	NT	7.00–45.10
Vitamin B12 (pmol/L)	NT	304.00	NT	NT	NT	NT	NT	133.00–675.00
Serum ferritin (ng/mL)	NT	1,561.00	NT	NT	NT	NT	NT	23.90–336.20
EPO (mIU/mL)	NT	56.87	NT	NT	NT	NT	NT	2.59–18.50
Irregular red cell antibodies	Negative	3+	Negative	Negative	Negative	Negative	Negative	Negative
DAT for anti-IgG	Negative	Negative	Negative	Negative	Negative	Negative	Negative	Negative
DAT for anti-C3d	Negative	3+	3+	3+	3+	2+	1+	Negative

RBC, red blood cells; MCV, mean corpuscular volume; MCH, mean corpuscular hemoglobin; MCHC, mean red blood cell hemoglobin concentration; LDH, lactate dehydrogenase; EPO, erythropoietin; DAT, direct anti-globulin test; NT, not tested.

## Diagnosis based on serological tests

Venous blood samples were collected at 3 (27 November 2021), 4 (4 December 2021), 6 (18 December 2021), 8 (31 December 2021), 11 (21 January 2022), and 14 weeks (11 February 2022) post-chemotherapy of pemetrexed plus cisplatin. Serological analysis of the patient's blood samples was performed, including DAT for anti-IgG and anti-C3d, acid elution test, and irregular RBC antibody screening IAT in the Coombs card. The methods and materials used are described in detail in the Supplementary material.

Direct antiglobulin test results for anti-C3d were positive for all the blood samples collected from 3 to 14 weeks after chemotherapy of pemetrexed plus cisplatin but were negative for anti-IgG. However, the reaction of DAT for anti-C3d became weaker over time from 8 to 14 weeks after chemotherapy. The results of irregular RBC antibody screening were positive at 3 weeks but negative at 4 and 6 weeks after the chemotherapy. Detailed results of DAT and irregular RBC antibody screening are presented in Table 1.

### Testing in the presence of soluble drugs

The patient's serum was incubated with untreated and enzyme-treated RBCs in a solution containing pemetrexed or cisplatin. The results of the tests performed using the drug solution are summarized in Table 2. The patient's serum reacted only with enzyme-treated RBCs in the presence of both pemetrexed and fresh sera from 3 to 8 weeks after chemotherapy. This result indicates that anti-pemetrexed antibodies may be present in the patient's serum and that the response may require the involvement of complement. However, pemetrexed-induced antibodies disappeared after 11-week post-chemotherapy. This indicates that antibodies were gradually cleared from the patient's circulation after a single dose of this drug. Interestingly, the patient's serum and normal sera reacted with both untreated and enzyme-treated RBCs with cisplatin from 3 to 14 weeks post-chemotherapy. These results may be due to the occurrence of NIPA in the presence of cisplatin.

**Table 2:** Testing the reaction in the presence of soluble drugs.

**Sample tested**	**Pemetrexed or PBS (3, 4, 6, 8 weeks post-chemotherapy)**		**Pemetrexed or PBS (11, 14 weeks post-chemotherapy)**		**Cisplatin or PBS (3, 4, 6, 8, 11, 14 weeks post-chemotherapy)**	
	**Untreated RBCs**	**Enzyme-treated RBCs**	**Untreated RBCs**	**Enzyme-treated RBCs**	**Untreated RBCs**	**Enzyme-treated RBCs**
Patient's serum + drug	0	0	0	0	2+	3+
Patient's serum + PBS	0	0	0	0	0	0
Patient's serum + C + drug	0	1+	0	0	2+	3+
Patient's serum + C + PBS	0	0	0	0	0	0
Normal sera + drug	0	0	0	0	2+	3+
Normal sera + PBS	0	0	0	0	0	0

PBS, phosphate buffer saline; C, compliment; RBC, red blood cell.

### Testing of drug-treated RBCs

Table 3 presents the reaction of the patient's serum pooled normal sera to drug-treated RBCs. The test results on all follow-up dates were identical. The patient's serum did not react with pemetrexed-treated RBCs. The patient's serum, diluted serum, and normal sera were all reactive with cisplatin-coated RBCs. In contrast, elution prepared from the patient's RBCs and diluted normal sera failed to react with cisplatin-coated RBCs. Combined with the previously mentioned results of the reaction with cisplatin, as presented in Table 2, it is suggested that NIPA may be present on the membrane of cisplatin-treated RBCs.

**Table 3:** Testing the reaction with drug-treated RBCs.

**Sample tested**	**Pemetrexed-treated RBCs (3, 4, 6, 8, 11, 14 weeks post-chemotherapy)**	**Cisplatin-treated RBCs (3, 4, 6, 8, 11, 14 weeks post-chemotherapy)**	**Untreated RBCs (3, 4, 6, 8, 11, 14 weeks post-chemotherapy)**
Patient's serum	0	2+	0
Patient's serum diluted 1 in 20	0	1+	0
Eluate	0	0	0
Last wash	0	0	0
Normal sera	0	2+	0
Normal sera diluted 1 in 20	0	0	0
PBS	0	0	0

PBS, phosphate buffer saline; RBC, red blood cell.

The reason that the patient's serum, diluted serum, and normal sera all consistently reacted with RBCs in the presence of soluble cisplatin, as well as with cisplatin-treated RBCs, may be that the concentration of cisplatin added in *in vitro* serological tests is much higher than that *in vivo*. Although the intensity of the DAT reaction weakens over time, implying that cisplatin-associated NIPA also weakens over time, less and less cisplatin gets adsorb nonspecifically to the RBC membrane. Therefore, using serological methods, we confirmed that pemetrexed and cisplatin might cause DIIHA in two distinctive ways.

## Assessment, treatment modification, and follow-up

According to the clinical symptoms, laboratory test results, and related medication history of the patient, the diagnosis of DIIHA was made. Due to severe anemia, the patient received two units of suspended RBCs on 7 November 2021. Then, the chemotherapy regimen of pemetrexed plus cisplatin was discontinued. The patient was not treated with glucocorticoids. For further treatment of lung adenocarcinoma, the chemotherapy regimen (every 3 and 4–6 weeks) was converted to albumin-bound paclitaxel (200 mg/m^2^, Day 1) plus carboplatin (Area under Curve 5, Day 1) from 2 December 2021. The level of HGB gradually increased, and the level of Rets, IBIL, and LDH returned to normal. The timeline of the patient's major clinical symptoms, the entire course of the disease, and therapy is demonstrated in Figure 1.

## Discussion

The diagnosis of drug-induced immune hemolytic anemia requires a definite manifestation of hemolytic anemia, a temporal relationship to drug administration, a positive DAT after drug application, and an attenuated hematologic reaction in the patient's serum after drug cessation (6, 12). The patient was reported to have clinical features such as immune hemolysis, negative eluate, positive DAT, presence of pemetrexed-induced complement-dependent antibodies, cisplatin-associated NIPA, and a gradual recovery from anemia, supporting the diagnosis of DIIHA.

Anemia is one of the most frequent side effects of chemotherapy. The two leading causes of anemia are insufficient RBC production due to bone marrow suppression and excessive RBC destruction due to various reasons. Pemetrexed is a dose-dependent, multitargeted folate analog that suppresses tumor growth by impeding DNA synthesis and folate metabolism (13). Supplementary folic acid and vitamin B12 during chemotherapy reduce RBC toxicities while maintaining antitumor activity (14, 15). The antineoplastic activity of cisplatin is mainly due to its ability to cross-link with DNA, thereby blocking transcription and replication (16). Cisplatin-based therapy often has a disproportionate effect on erythropoiesis compared to other blood cells, leading to a cumulative, clinically significant anemia (17). In addition, long-term application of cisplatin may lead to renal tubular damage, resulting in a decreased level of erythropoietin (EPO) produced by peritubular interstitial cells, leading to renal anemia (18, 19). Although both pemetrexed and cisplatin can affect erythropoiesis, the process is chronic and cumulative in its onset. Our patient was supplemented with folic acid and vitamin B12 before chemotherapy. The patient had a normal renal function and urinalysis pre- and post-chemotherapy and was adequately hydrated during chemotherapy to reduce renal toxicity. Moreover, the average lifespan of erythrocytes is about 120 days. Thus, a decrease in erythropoiesis due to bone marrow suppression or EPO reduction after the first cycle of chemotherapy with pemetrexed plus cisplatin is unlikely.

Pemetrexed-induced antibodies were detected in the patient's serum. Moreover, the reaction is complement-dependent, as confirmed by positive anti-C3d in DAT. These results were similar to those in previous cases of pemetrexed-induced DIIHA in the treatment of non-small cell lung cancer. In one report, the DAT test was positive for anti-C3d (20), while in another report, the DAT test was positive for both anti-IgG and anti-C3d (21). Antibodies are probably IgM, and there is a possible secondary switch to IgG, IgA, or even IgE through a recombination/deletion process termed immunoglobulin heavy chain class switch recombination (22, 23). The unique feature of our patient is that pharmacologic antibodies induced by pemetrexed were complement-dependent. The C3d component is the inactivated form of C3b, which is involved in phagocytosis, formation of C3 convertase, and stimulation of complement (10, 24, 25). C3d-coated RBCs have helped us understand that cells survive complement activation either by the classical pathway, i.e., due to drug-dependent antibodies to RBCs or due to activations of an alternative pathway in the presence of nearby RBCs (6). We speculate that the immune complex formed by the antibody-pemetrexed-complement-RBC membrane results in hemolysis.

Non-immunologic protein adsorption onto the RBC membrane has been observed for many drugs, including cisplatin. These drugs have a β-lactam moiety to bind to RBCs *via* a chemical moiety that is not a β-lactam moiety, resulting in exposure of β-lactam moiety that covalently binds to other proteins (immunoglobulins, complement, album, etc.) (26). Absorbed proteins can react with receptors on macrophages and cause RBC destruction though they are not antibodies against drugs or RBCs. Although it is not an antibody-mediated process, RBCs get damaged due to a distinct immune response, similar to RBCs coated with drugs and drug-specific antibodies (27). Two previous studies investigated that cisplatin-associated DIIHA was due to NIPA instead of cisplatin-induced antibodies (8, 28). In addition, the pooled normal sera had a reaction on IAT, but the normal sera diluted 1 in 20 did not react, which is consistent with our study.

The essential treatment of DIIHA is to stop using the suspected drug. Transfusion and plasma exchange may be necessary in some severe cases (4, 29, 30). The role of glucocorticoids in DIIHA treatment remains unclear (4). In our case, we promptly administered a blood transfusion, and the patient gradually recovered from anemia after discontinuation of pemetrexed and cisplatin. Considering the effect of glucocorticoids on lung cancer, we did not apply glucocorticoids to the patients. In addition, a folic acid supplement is necessary for patients with hemolytic anemia, especially in patients with chronic hemolysis. Our patient had started folic acid supplement therapy before chemotherapy, but the dose used was 400 μg/day, which is lower than the recommended supplement dose in patients with hemolytic anemia (1,000 μg/day). Considering the acute onset of DIIHA, we did not adjust the folic acid supplementation dose, but the patient's folic acid supplementation was not stopped until 2 months after discontinuation of pemetrexed chemotherapy, by which time the patient's anemia had significantly improved.

Tube testing is a classic method of detecting drug-dependent or drug-independent antibodies. The advantages of tube testing are its convenience, rapidness, and low expenses. In previous studies, most scholars used the tube method for DIIHA-related serological testing. In 2020, Tan Ngoc Nguyen and his team confirmed that the gel method was more sensitive, reliable, and reproducible than the conventional tube method for detecting all drug-dependent antibodies investigated in their studies (26, 31). Hence, we performed all serological tests using the gel method.

In summary, we reported a case of DIIHA that may be induced by complement-dependent pemetrexed antibodies and cisplatin-induced NIPA. To our knowledge, this is the first case of the two possible mechanisms of DIIHA in a single patient. Although DIIHA is rare, it is a potentially life-threatening complication that requires careful monitoring when chemotherapy or other medications are administered.

## Data availability statement

The original contributions presented in the study are included in the article/Supplementary material, further inquiries can be directed to the corresponding authors.

## Ethics statement

The studies involving human participants were reviewed and approved by the Ethics Committee of China-Japan Friendship Hospital. The patients/participants provided their written informed consent to participate in this study.

## Author contributions

HLu, NW, and SW analyzed and interpreted the patient data and were the major contributors to writing the manuscript. PW, RZ, and HLiu performed interpretation of serological tests. XH, ZL, and YCh collected the blood samples and conducted the related laboratory tests. HZ and YCa revised the manuscript. All authors contributed to the article and approved the submitted version.

## Conflict of interest

The authors declare that the research was conducted in the absence of any commercial or financial relationships that could be construed as a potential conflict of interest.

## Publisher's note

All claims expressed in this article are solely those of the authors and do not necessarily represent those of their affiliated organizations, or those of the publisher, the editors and the reviewers. Any product that may be evaluated in this article, or claim that may be made by its manufacturer, is not guaranteed or endorsed by the publisher.
