# Supplementary material for: Case report: First case of pemetrexed plus cisplatin-induced immune hemolytic anemia in a patient with lung adenocarcinoma
Source: Front Med (Lausanne). 2022 Aug 25;9:917485. doi: 10.3389/fmed.2022.917485 (PMC9452692; doi:10.3389/fmed.2022.917485)
Supplement: Supplementary file 1 [file Presentation_1.pdf]

Materials and Methods

Serological tests were performed according to procedures outlined in the AABB technical Manual with minor changes. All the serological tests were carried out with standard test gel techniques. DAT was conducted with monospecific gel cards (anti-IgG or anti-C3d) (LiBiotech, Wuxi, China), and other indirect antiglobulin tests with or without drug were carried out with polyspecific gel cards (anti-IgG or anti-C3d) (Diagnostic Grifols, Barcelona, Spain). An acid eluate was prepared from the patient's RBCs by using a commercial elution kit (Baso diagnostics, Inc., Zhuhai, China). The patient's serum (collected 3 weeks, 4 weeks, and 6 weeks post-drug intake) was tested for antibodies to pemetrexed and cisplatin by two methods:1) in the presence of soluble drug against untreated and enzyme-treated RBCs and 2) against drug-treated RBCs. Pemetrexed (Hansoh Pharmaceutical Co.,Ltd, Jiangsu, China) and cisplatin (Qilu Pharmaceutical Co.,Ltd, Jinan, China) were supplied as 1 mg/ml solution(1, 2).

Testing in the presence of soluble drug

25µL of patient's serum and 25µL of pemetrexed or cisplatin solution, with and without fresh normal serum as a source of complement, were incubated with 50µL 1% untreated (Diagnositic Grifols, Barcelona, Spain) or ficinase-treated RBCs (Sanquin Reagents, Amsterdam, Netherland) at 37°C for 1hour. Control groups with PBS substituted for drug solution and normal sera (pool of three healthy individuals' sera, negative antibody screen test) substituted for patient's serum were tested in parallel.

Preparation and testing of drug-treated RBCs

Drug-treated RBCs were prepared by incubating 0.1ml group O RBCs with 1ml of the drug solution for 1hour at 37°C. Both pemetrexed and cisplatin were used at 1 mg/ml concentration in PBS based on previous studies(1, 2).

References

1. Leger RM, Arndt PA, Garratty G. How we investigate drug-induced immune hemolytic anemia. *Immunohematology*. (2014) 30:85-94

2. Nguyen TN, Fihman V, Maenulein E, Vinatier I, Klaren JM. Drug-induced immune hemolytic anemia investigation: Comparison between tube test and microcolumn agglutination (gel test) for the detection of drug-dependent antibodies in the presence of soluble drug. *Transfus Clin Biol*. (2020) 27:133-8. doi:10.1016/j.tracli.2020.06.003
